# Clinical characteristics and outcomes of patients admitted to hospitals for posterior reversible encephalopathy syndrome: a retrospective cohort study

**DOI:** 10.1186/s12883-021-02143-6

**Published:** 2021-03-09

**Authors:** Abbas Alshami, Asseel Al-Bayati, Steven Douedi, Mohammad A. Hossain, Swapnil Patel, Arif Asif

**Affiliations:** Department of Medicine, Jersey Shore University Medical Center, Neptune, NJ 07753 USA

**Keywords:** Posterior reversible encephalopathy syndrome, Neurologic complications, Cerebral hemorrhage, Seizures

## Abstract

**Background:**

Posterior reversible encephalopathy syndrome (PRES) is usually a benign, yet underdiagnosed clinical condition associated with subacute to acute neurological manifestations primarily affecting white matter. PRES is reversible when recognized promptly and treated early by removal of the insulting factor; however, can lead to irreversible and life-threatening complications such as cerebral hemorrhage, cerebellar herniation, and refractory status epilepticus.

**Methods:**

We utilized the National Inpatient Sample database provided by the Healthcare Cost and Utilization Project (HCUP-NIS) 2017 to investigate the demographic variables (age, sex, and race) for patients with PRES, concomitant comorbidities and conditions, inpatient complications, inpatient mortality, length of stay (LOS), and disposition.

**Results:**

A total of 635 admissions for patients aged 18 years or older with PRES were identified. The mean age was 57.2 ± 0.6 years old with most encounters for female patients (71.7%, *n* = 455) and white as the most prevalent race. Half the patients in our study presented with seizures (50.1%, *n* = 318), sixty-three patients (9.9%) presented with vision loss, and sixty-four patients (10.1%) had speech difficulty. In addition, 45.5% of patients had hypertensive crisis (*n* = 289). 2.2% of hospitalizations had death as the outcome (*n* = 14). The mean LOS was 8.2 (±0.3) days, and the mean total charges were $92,503 (±$5758). Inpatient mortality differed between males and females (1.7% vs. 2.4%) and by race (3.6% in black vs. 1.8% in white) but was ultimately determined to be not statistically significant. Most patients who present with vision disturbance have a high risk of intracranial hemorrhage. Furthermore, end-stage renal disease, atrial fibrillation, and malignancy seemed to be linked with a very high risk of mortality.

**Conclusion:**

PRES, formerly known as reversible posterior leukoencephalopathy, is a neurological disorder with variable presenting symptoms. Although it is generally a reversible condition, some patients suffer significant morbidity and even mortality. To the best of our knowledge, this is the largest retrospective cohort of PRES admissions that raises clinician awareness of clinical characteristics and outcomes of this syndrome.

## Introduction

Posterior reversible encephalopathy syndrome (PRES) is a clinical, radiographic syndrome ranging from subacute to acute neurological manifestations primarily affecting the white matter [1, 2]. PRES has been associated with hypertension, eclampsia, chemotherapeutic and immunosuppression medications, and transplant-recipient patients [1, 2]. Generally, it is considered a benign disease if recognized early and promptly treated with the removal of causative factors [3, 4]. However, there is a myriad of clinical conditions that when manifest with PRES symptoms carry a poor prognosis, such as life-threatening cerebral hemorrhage, cerebellar herniation, and refractory seizures.

PRES is still an underdiagnosed neurological syndrome with a constellation of symptoms that share characteristic neuroimaging findings [2, 3]. Demographically, it commonly affects middle-aged females with no statistical significance in mortality, gender, or race [5]. The most common symptoms include headache, confusion, visual disturbance, altered mental status, and seizures [2, 3, 6]. Neuroimaging shows a distinctive parieto-occipital finding in a bi-hemispheric distribution that reflects vasogenic edema [2, 3].

PRES is reversible when recognized early and treated promptly by removal of the insulting factor; nonetheless, it can lead to irreversible and life-threatening complications which include cerebral hemorrhage, cerebellar herniation, and refractory status epilepticus [7]. In this analysis, we sought to investigate the clinical characteristics and outcomes of patients admitted to hospitals for PRES. Additionally, we aimed to identify predictors of unfavorable outcomes in relation to this syndrome.

## Methods

### Study design/settings

We utilized the National Inpatient Sample database provided by the Healthcare Cost and Utilization Project (HCUP-NIS) 2017. This database is a weighted 20% sample of all-payer hospitalizations in the United States. The selection of these hospitalizations was made systematically by the Agency for Healthcare Resources and Quality (AHRQ) to represent all the hospitalizations in the United States in 2017. The data collected includes demographic variables, admission diagnoses, procedures, type of insurance, geographical location, length of stay, inpatient mortality, and disposition, among others. These encounters represent hospitalizations, not patients, so multiple potential hospitalizations for the same patient are considered as multiple encounters.

### Participants

We included all hospitalizations for patients primarily admitted for PRES. Encounters for patients aged < 18 years old were excluded from this study.

### Variables

We aimed to investigate the demographic variables (age, sex, and race) for these patients, concomitant comorbidities and conditions, inpatient complications, inpatient mortality, length of stay (LOS), and disposition.

### Data measurement

PRES and concomitant comorbidities and complications were identified through their international classification of diseases, 10th revision (ICD-10 codes) recorded in the discharge record for each hospitalization.

### Ethical considerations

The NIS databases are considered “limited data sets” by the HIPAA privacy regulations; thus, do not require revision by an institutional review board (IRB) (http://privacyruleandresearch.nih.gov). The study was conducted in agreement with the principles of the Declaration of Helsinki.

### Statistical methods

Continuous and categorical variables were described as mean with standard deviation and frequencies, as appropriate. Chi-square was used to compare categorical variables, and an independent sample *t-test* was used to compare continuous variables. Multivariate logistic regression models (Enter method) were used to predict outcomes, and Hosmer and Lemeshow (HL) test were used to assess goodness of fit. Variables that predicted outcomes in univariate analyses (*p* < 0.1) were included in the multivariate models. Correlation matrices were created and evaluated to rule out any potential multicollinearities. All analyses were done using IBM SPSS statistics version 26.0 (IBM Corporation, Artmonk, NY). An alpha value (p) of 0.05 was used to ascertain statistical significance.

## Results

A total of 635 admissions for PRES were identified. Missing data were only 26 values in race variable and three values in primary expected payer. All analyses were done using pairwise deletion for missing data, as missingness of less than 5% of data is unlikely to introduce bias [8].

### Demographic variables

The mean age was 57.2 ± 0.6 years old. Most encounters were for female patients (71.7%, *n* = 455). White was the most prevalent race. Medicare, followed by Medicaid, were the most common primary payers (Table 1).

**Table 1:** Demographic characteristics of admitted patients

		Mean (SD)	Frequency (%)
Age (years)		57.2 (15.4)	61.8 (17.2)
Sex	Male		180 (28)
	Female		455 (72)
Race	White		435 (68.5)
	Black		112 (17.6)
	Hispanic		36 (5.7)
	Asian or Pacific Islander		7 (1.1)
	Native American		2 (0.3)
	Other		17 (2.7)
Primary expected payer	Medicare		312 (49.1)
	Medicaid		123 (19.4)
	Private insurance		151 (23.8)
	Self-pay		32 (5)
	No charge		2 (0.3)
	Other		12 (1.9)
Comorbidities/Conditions	Hypertension		549 (86.5)
	Diabetes Mellitus		186 (29.3)
	Dehydration		90 (14.2)
	Hyperlipidemia		219 (34.5)
	Depression		123 (19.4)
	Anxiety		133 (20.9)
	Smoking		116 (18.3)
	Hypothyroidism		98 (15.4)
	GERD		135 (21.3)
	Malnutrition		67 (10.6)
	CKD		115 (18.1)
	ESRD		108 (17)
	Atrial fibrillation		64 (10.1)
	Rheumatological disease		47 (7.4)
	Obesity		89 (14)
	Malignancy		82 (12.9)
	Infection		198 (31.2)
	Migraine		36 (5.7)
	Coagulopathy		45 (7.1)
	Thrombocytopenia		44 (6.9)
	History of Organ Transplant		30 (4.7)
Total			635 (100)

*Abbreviations*: *GERD* gastroesophageal reflux disease, *CKD* chronic kidney disease, *ESRD* end-stage renal disease

### Presenting signs and symptoms

Half the patients presented with seizures (50.1%, *n* = 318), sixty-three patients (9.9%) presented with vision loss, and sixty-four patients (10.1%) had speech difficulty. In addition, 45.5% of patients had hypertensive crisis (*n* = 289).

### Outcomes

2.2% of hospitalizations had death as the outcome (*n* = 14). Mean LOS was 8.2 (±0.3) days, and the mean total charges were $92,503 (±$5758). Inpatient mortality differed between males and females (1.7% vs. 2.4%); however, the difference was not statistically significant (*p* = 0.561). Mortality also differed by race (3.6% in black vs. 1.8% in white), but none of the differences were statistically significant (*p* = 0.786). Similarly, there was no statistical difference in length of stay between males and females. Ninety-one patients (14.3%) were documented to have cerebral edema. Twenty-nine patients developed intracranial hemorrhage (4.6%), while 32 patients developed cerebral infarction (5%). Regarding disposition out of the hospital, 67.4% (*n* = 428) were discharged home, while 18.22% (*n* = 115) were transferred to other facilities such as skilled nursing facility, intermediate care facility, inpatient rehabilitation, long term care hospital, among others, not including patients transferred to the hospital from other facilities.

### Predictors of unfavorable outcomes

The predictability of different variables for unfavorable outcomes (mortality, cerebral infarction, intracranial hemorrhage) was evaluated using univariate analyses (Table 2). The presence of anemia, infection, malignancy, atrial fibrillation, difficulty speaking, end stage renal disease (ESRD), malnutrition, and coagulopathy predicted mortality (*p* < 0.1) in univariate analyses. In a multivariate regression model only malignancy, atrial fibrillation, and ESRD predicted mortality (Table 3) and the HL test showed a good fit (*p* = 0.88). Vision loss and hypertension predicted intracranial hemorrhage in univariate analyses (*p* < 0.1). However, only vision loss predicted intracranial hemorrhage (OR 3.76 (95% CI 1.59–8.93, *p* = 0.003), with HL showing a good fit (*p* = 0.97). ESRD and dehydration predicted cerebral infarction in univariate analyses (*p* < 0.1) although none of them predicted it in a multivariate logistic regression model.

**Table 2:** Predictability of different variables for outcomes in univariate analyses

	Mortality	Cerebral infarction	ICH
Age	No	No	No
Sex	No	No	No
Race	No	No	No
Anemia	Yes	No	No
Infection	Yes	No	No
Malignancy	Yes	No	No
Diabetes Mellitus	No	No	No
Seizures	No	No	No
Vision loss	No	No	Yes
Obesity	No	No	No
Rheumatological disease	No	No	No
CKD	No	No	No
Atrial fibrillation	Yes	No	No
Speech difficulty	Yes	No	No
ESRD	Yes	Yes	No
Malnutrition	Yes	No	No
Migraine	No	No	No
Hypothyroidism	No	No	No
Smoking	No	No	No
Depression	No	No	No
Anxiety	No	No	No
Hypertension	No	No	Yes
Hypertensive crisis	No	No	No
Hyperlipidemia	No	No	No
Coagulopathy	Yes	No	No
Dehydration	No	Yes	No
Thrombocytopenia	No	No	No
History of organ transplantation	No	No	No

*Abbreviations*: *ICH* intracranial hemorrhage, *CKD* chronic kidney disease, *ESRD* end-stage renal disease

**Table 3:** Multivariate logistic regression model to predict mortality

	OR	95% CI for OR	*p*-Value
Anemia	0.933	0.25–3.48	0.917
Infection	3.113	0.95–10.23	0.061
Malignancy	4.187	1.07–16.32	0.039
Atrial fibrillation	3.842	1.01–14.55	0.048
ESRD	7.270	2.06–25.63	0.002
Malnutrition	2.201	0.58–8.36	0.246
Coagulopathy	2.293	0.54–9.83	0.264

*Abbreviations*: *ESRD* end-stage renal disease, *OR* odds ratio, *CI* confidence interval

## Discussion

At present, the exact pathophysiology of PRES unknown; however, multiple theories have centered around the disruption of the blood-brain barrier (BBB) by an insulting factor leading to extravasation of intravascular fluid and cerebral edema [9]. Rapid elevation of blood pressure is well known to disrupt the BBB and has been noted in most reported cases of PRES [6, 10]. Other causes of BBB disruption include autoimmune diseases, which were also noted in patients with PRES, especially those with normal blood pressure [11]. Endothelial damage can also be cytokine-mediated [11]. Cytokines stimulate the secretion of vasoactive factors such as vascular endothelial growth factor (VEGF), which increases permeability. The following discusses a few important prognostic indicators and factors that impact patient outcomes.

### Demographics

PRES can present at any age and can range widely. Our average age of presentation for our patients was 57 years old; however, cases have been reported as old as 90 years old [3]. PRES is most commonly seen in women than in men. Our study demonstrated similar results, with 72% of PRES patients being females. It has been proposed previously that women may be a higher risk and have more severe symptoms which may be due to women having fewer inter-neuronal connections and more diffusivity in parieto-occipital regions [12, 13].

### Clinical presentation

Presenting symptoms of our patients were consistent with those observed in prior studies. The main symptoms included seizures and hypertension [2, 3, 6]. Our study validates that hypertension may play a key role in the development of PRES as about 87% of admitted patients were hypertensive. This was explained in previous studies by the direct effect of blood pressure on the blood-brain barrier function and the resultant brain edema [14]. In an animal model, a rapid elevation of blood pressure resulted in leakage or exudation of plasma, macromolecules, or red blood cells [15].

Vision disturbance was also reported in 63 patients (9.9%). This relates to the occipital lobe involvement that gives various presentations such as vision loss and visual hallucinations [16, 17]. Our study revealed a strong correlation between vision loss, among all other presentations, with intracranial hemorrhage (OR 3.76, *p* = 0.003). The pathogenesis of parenchymal or sulcal subarachnoid hemorrhage in PRES could be due to either post-ischemic reperfusion damage or impaired autoregulation [18, 19]. Speech difficulty was reported in 64 patients (10.1%).

### PRES and ESRD

In our study, ESRD was a high predictor of mortality (*n* = 108 [17%]) in patients with PRES, with the likelihood of dying about seven times higher than those without ESRD (OR 7.27, 95% CI 2.06–25.63, *p* = 0.002). Many studies revealed a heterogeneous association of renal diseases with PRES, ranging from no association in one series to approximately 30% in other large series [6, 20]. Several possible etiological factors play a role in the pathogenesis including hypertension, drugs, and fluid/electrolyte disturbances. ESRD patients have a higher likelihood of hypertension and fluid overload, which supports the hyperperfusion theory of PRES in ESRD patients [21, 22]. In these patients, toxic metabolic effects of urea may lead to cytokine-mediated damage to the blood-brain barrier. Another possible mechanism is vasoconstriction from erythropoietin infusion [23]. There have also been reports of PRES in patients who are started on hemodialysis [24]. It is hypothesized that initiation of dialysis results in a difference in pre-treatment and post-treatment urea levels and resultant acidosis. This promotes the shift of sodium, potassium, and other electrolytes which contributes to endothelial dysfunction and resultant vasogenic edema [21, 25]. Our study did not review patients who were initiated on dialysis; however, it still does show a strong correlation between ESRD and mortality in PRES patients. Patients with ESRD and PRES should be monitored closely due to the higher chance of mortality.

### Malignancies

Our study found malignancy to be a strong predictor of mortality in PRES (OR 4.18, 95% CI 1.07–16.32, *p*-value = 0.039), which was consistent with prior studies [25, 26]. Cancer patients are more likely to have other comorbidities than non-cancer patients, such as thrombocytopenia, renal failure, or medication-induced hypertension [26]. Immunosuppressive and chemotherapeutic agents have another potential etiology for the increased incidence of PRES in these patients. The neurotoxicity of these agents is well known in the literature but still not yet fully understood [10]. Toxic levels in the blood are not required to develop PRES and can develop at any time during therapy [10]. Few studies have reported complete resolution of the clinical and radiological symptoms despite the continuation of medications [27]. Because of the markedly increased mortality in cancer patients who develop PRES, we stress early measures to diagnose and treat these patients when there is a slight suspicion of PRES. Early lowering of the drug dose or complete discontinuation of the cytotoxic or immunosuppressive drug is recommended to prevent deterioration of the clinical condition, avoiding permeant deficit and poor outcomes [26].

### Atrial fibrillation

Atrial fibrillation was documented in approximately 10% of our patients with a odds ratio suggesting a four time higher risk of mortality (OR 3.842, 95% CI 1.01–14.55, *p*-value = 0.048). A prior study by Hinduja et al. examining patients with PRES and predictability of intensive care unit admissions showed that all patients with atrial fibrillation required ICU admission [28]. This implied the correlation that patients with PRES and atrial fibrillation may be more critically ill.

### Sepsis and severe infections

In our study, 198 patients (31.2%) had an associated severe infection or sepsis. These patients had a high odds ratio or mortality; however, this did not reach statistical significance (OR 3.1, 95%CI 0.95–10.23, *p* = 0.061). Severe infection or sepsis association with PRES was previously reported in the literature; however, was not well established as other comorbidities (e.g., hypertension, pre-eclampsia/eclampsia, cytotoxic/immunosuppressive, and autoimmune disease) [29]. Many theories have been put forward to explain the pathophysiology related to this association. As an example, Marra et al. described the mechanism of endothelial damage of PRES in sepsis patients by the primary inflammatory involvement with T cell activation and cytokine release [9]. Separately, Gao et al. in their neuropeptide theory suggested that endothelial dysfunction is induced by the release of endothelin-1, prostacyclin, and thromboxane A2 [30]. Powerful stimulation of the systemic inflammatory response may be related to the development of PRES in severe infection [31, 32]. Further investigations are needed accurately to determine a possible correlation between PRES and infections and its impact on mortality.

## Conclusion

Posterior reversible encephalopathy syndrome (PRES), formerly known as reversible posterior leukoencephalopathy, is a neurological disorder with variable presenting symptoms. Although it is generally a reversible condition, some patients suffer significant morbidity and even mortality. Upon reviewing the literature to date, this is the most extensive retrospective study of PRES admissions. Most patients who present with vision disturbance have a high risk of intracranial hemorrhage. Furthermore, ESRD, atrial fibrillation, and malignancy seemed to be linked with a very high risk of mortality. Ours is the largest study that raises awareness of PRES among the internists, neurologists, nephrologists, and hemato-oncologist and calls for a collaborative approach to mitigate the devastating consequences of this syndrome. Based upon the findings, we suggest prompt and meticulous fluid/electrolyte balance, blood pressure control, and avoidance of rapid changes from baseline, and chemotherapeutic agents in the management and reversal of this condition. Since kidney injury can be an important determinant of outcomes, further studies are needed to determine the impact and degree of renal insufficiency on PRES.

## Data Availability

The authors declare that data supporting the findings of this study are available within the article.

## Abbreviations

PRES: Posterior reversible encephalopathy syndrome
LOS: Length of stay
HCUP: Healthcare Cost and Utilization Project
NIS: National Inpatient Sample
OR: Odds Ratio
CI: Confidence interval
ICH: Intracranial hemorrhage
CKD: Chronic kidney disease
ESRD: End stage renal disease
BBB: Blood-brain barrier

## References

[1] Hossain MA, Jehangir W, Nai Q (2015). Posterior reversible encephalopathy syndrome in a bone marrow transplant patient: a complication of immunosuppressive drugs?. World J Oncol.

[2] Yoon SD, Cho BM, Oh SM, Park SH, Jang IB, Lee JY (2013). Clinical and radiological spectrum of posterior reversible encephalopathy syndrome. J Cerebrovasc Endovasc Neurosurg.

[3] López-García F, Amorós-Martínez F, Sempere AP (2004). A reversible posterior leukoencephalopathy syndrome. Rev Neurol.

[4] Lee VH, Wijdicks EFM, Manno EM, Rabinstein AA (2008). Clinical spectrum of reversible posterior leukoencephalopathy syndrome. Arch Neurol.

[5] Fischer M, Schmutzhard E (2017). Posterior reversible encephalopathy syndrome. J Neurol.

[6] Fugate JE, Claassen DO, Cloft HF, Kallmes DF, Kozak OS, Rabinstein AA (2010). Posterior reversible encephalopathy syndrome: associated clinical and radiologic findings. Mayo Clin Proc.

[7] Cordelli DM, Masetti R, Ricci E (2014). Life-threatening complications of posterior reversible encephalopathy syndrome in children. Eur J Paediatr Neurol.

[8] Dong Y, Peng CY (2013). Principled missing data methods for researchers. Springerplus..

[9] Marra A, Vargas M, Striano P, Del Guercio L, Buonanno P, Servillo G (2014). Posterior reversible encephalopathy syndrome: the endothelial hypotheses. Med Hypotheses.

[10] Covarrubias DJ, Luetmer PH, Campeau NG (2002). Posterior reversible encephalopathy syndrome: prognostic utility of quantitative diffusion-weighted MR images. Am J Neuroradiol.

[11] Bartynski WS, Boardman JF (2007). Distinct imaging patterns and lesion distribution in posterior reversible encephalopathy syndrome. Am J Neuroradiol.

[12] Schmithorst VJ, Holland SK, Dardzinski BJ (2008). Developmental differences in white matter architecture between boys and girls. Hum Brain Mapp.

[13] Ducros A, Fiedler U, Porcher R, Boukobza M, Stapf C, Bousser MG (2010). Hemorrhagic manifestations of reversible cerebral vasoconstriction syndrome: frequency, features, and risk factors. Stroke..

[14] Li Y, Gor D, Walicki D, Jenny D, Jones D, Barbour P, Castaldo J (2012). Spectrum and potential pathogenesis of reversible posterior leukoencephalopathy syndrome. J Stroke Cerebrovasc Dis.

[15] Häggendal E, Johansson B (1972). On the pathophysiology of the increased cerebrovascular permeability in acute arterial hypertension in cats. Acta Neurol Scand.

[16] Bakshi R, Bates VE, Mechtler LL, Kinkel PR, Kinkel WR (1998). Occipital lobe seizures as the major clinical manifestation of reversible posterior leukoencephalopathy syndrome: magnetic resonance imaging findings. Epilepsia..

[17] Tajima Y, Isonishi K, Kashiwaba T, Tashiro K (1999). Two similar cases of encephalopathy, possibly a reversible posterior leukoencephalopathy syndrome: serial findings of magnetic resonance imaging, SPECT and angiography. Intern Med.

[18] Doss-Esper CE, Singhal AB, Smith MS, Henderson GV (2005). Reversible posterior leukoencephalopathy, cerebral vasoconstriction, and strokes after intravenous immune globulin therapy in Guillain-Barre syndrome. J Neuroimaging.

[19] Godasi R, Rupareliya C, Bollu PC (2017). Bilateral occipital lobe hemorrhages presenting as denial of blindness in posterior reversible encephalopathy syndrome- a rare combination of Anton syndrome and encephalopathy. Cureus..

[20] Gatla N, Annapureddy N, Sequeira W, Jolly M (2013). Posterior reversible encephalopathy syndrome in systemic lupus erythematosus. J Clin Rheumatol.

[21] Rizzo MA, Frediani F, Granata A, Ravasi B, Cusi D, Gallieni M (2012). Neurological complications of hemodialysis: state of the art. J Nephrol.

[22] Garg RK (2001). Posterior leukoencephalopathy syndrome. Postgrad Med J.

[23] Ermeidi E, Balafa O, Spanos G, Zikou A, Argyropoulou M, Siamopoulos KC (2013). Posterior reversible encephalopathy syndrome: a noteworthy syndrome in end-stage renal disease patients. Nephron Clin Pract.

[24] Ogawa A, Sugiyama H, Nakayama K, Morinaga H, Akagi S, Makino H (2012). Reversible posterior leukoencephalopathy syndrome in a young adult patient receiving peritoneal dialysis. Perit Dial Int.

[25] Seifter JL, Samuels MA (2011). Uremic encephalopathy and other brain disorders associated with renal failure. Semin Neurol.

[26] Kamiya-Matsuoka C, Paker AM, Chi L, Youssef A, Tummala S, Loghin ME (2016). Posterior reversible encephalopathy syndrome in cancer patients: a single-institution retrospective study. J Neuro-Oncol.

[27] Schwartz RB, Jones KM, Kalina P, Bajakian RL, Mantello MT, Garada B, Holman BL (1992). Hypertensive encephalopathy: findings on CT, MR imaging, and SPECT imaging in 14 cases. AJR Am J Roentgenol.

[28] Hinduja A, Habetz K, Raina SK, Fitzgerald RT (2017). Predictors of intensive care unit utilization in patients with posterior reversible encephalopathy syndrome. Acta Neurol Belg.

[29] Racchiusa S, Mormina E, Ax A, Musumeci O, Longo M, Granata F (2019). Posterior reversible encephalopathy syndrome (PRES) and infection: a systematic review of the literature. Neurol Sci.

[30] Gao B, Lyu C, Lerner A, McKinney AM (2018). Controversy of posterior reversible encephalopathy syndrome: what have we learnt in the last 20 years?. J Neurol Neurosurg Psychiatry.

[31] Bartynski WS, Boardman JF, Zeigler ZR, Shadduck RK, Lister J (2006). Posterior reversible encephalopathy syndrome in infection, sepsis, and shock. AJNR Am J Neuroradiol.

[32] Granata G, Greco A, Iannella G, Granata M, Manno A, Savastano E, Magliulo G (2015). Posterior reversible encephalopathy syndrome--insight into pathogenesis, clinical variants and treatment approaches. Autoimmun Rev.

